# Nurses retention: the impact of transformational leadership, career growth, work well-being, and work-life Balance

**DOI:** 10.1186/s12912-025-02762-1

**Published:** 2025-02-08

**Authors:** Eman Sameh AbdELhay, Samah Mohamed Taha, Mona Metwally El-Sayed, Sahar Hassan Helaly, Islam Sameh AbdELhay

**Affiliations:** 1 Assistant Professor of Psychiatric and Mental Health Nursing, Faculty of Nursing, Mansoura University, Mansoura, Egypt; 2 Assistant Professor of Psychiatric Nursing and Mental Health, Faculty of Nursing, Alexandria University, Alexanderia, Egypt; 3 Assistant Professor of Nursing Administration, Faculty of Nursing, Mansoura University, Mansoura, Egypt; 4 Lecturer of Nursing Administration, Faculty of Nursing, Mansoura University, Mansoura, Egypt

**Keywords:** Nurses retention, Transformational leadership, Career growth, Work well-being, Work-life balance

## Abstract

**Background:**

Nurse retention is critical for healthcare systems worldwide, as high turnover rates adversely affect patient care and organizational stability.

**Aim:**

Examines the impact of transformational leadership, career growth opportunities, work well-being, and work-life balance on nurse retention.

**Methods:**

A cross-sectional study was conducted with 297 nurses employed in inpatient wards at Mansoura University Hospital. The study measured variables using the Nursing Retention Index, Global Transformational Leadership Scale, Career Growth Scale, Work Well-Being Scale, and Work-Life Balance Scale.

**Results:**

A linear regression analysis identified significant predictors of nurse retention. Work-life balance was found to be a strong predictor, with an unstandardized coefficient (B) of 0.255 (*p* < 0.001) and a standardized coefficient (β) of 0.426, indicating a positive relationship with retention, transformational leadership also significantly influenced retention, with a B of 0.082 (*p* = 0.002) and a β of 0.171. In contrast, Career Growth and Work-Life Balance did not significantly predict retention, showing coefficients of -0.082 (*p* = 0.154) and -0.042 (*p* = 0.482). The model explained 23.4% of the variance in nurse retention (R-squared = 0.234) and demonstrated statistical significance (F = 22.294, *p* < 0.001).

**Conclusion:**

This study highlights the critical role of transformational leadership and work-life balance in enhancing nurse retention. However, career growth and work well-being did not significantly predict retention in this study. Healthcare institutions should focus on transformational leadership and work-life balance to enhance nurse retention. Future studies should explore factors related to career growth and work well-being to determine their potential impact on retention.

**Supplementary Information:**

The online version contains supplementary material available at 10.1186/s12912-025-02762-1.

## Introduction

The retention of nurses has emerged as a significant concern, given the alarming costs associated with nurse shortages [1, 2]. Retaining nurses has proven to be a challenge due to the potential for burnout among nursing staff, the adoption of various leadership styles by modern managers, and the misplacement of priorities, with an excessive focus on cost reduction rather than prioritizing the well-being of staff for long-term success [3–5]. Addressing this issue necessitates transformational leadership that is not solely focused on performance outputs but also demonstrates a genuine concern for nurse well-being—additionally, offering clear pathways for career growth that can contribute to an improved work-life balance [6, 7].

However, the health sector mainly needs help with human capital shortages, making it even more challenging to retain nurses in hospitals [8]. As a result, hospital managers are focusing on maintaining job satisfaction, improving job performance, and increasing nursing resources through strategies such as work-life balance to address the current shortage of human resources [9–11].

The round-the-clock nature of hospital operations has long been challenging for nursing staff, balancing work and family life. High workloads, sleep deprivation, and exhaustion can strain personal relationships with family and friends [12, 13]. Healthcare organizations need to prioritize the development of a work environment conducive to nurse retention, as this directly impacts the financial stability and quality of care provided [14–16]. The issue of nurse retention is a significant concern, with the definition evolving from simply maintaining staff levels to addressing staff retention issues, implementing long-term staffing strategies, and now expanding to include future employment and work design [17, 18].

Leadership impacts the ability to carry out tasks, influence subordinates, and enhance job satisfaction, and is also connected to the standard of patient care [19, 20]. Transformational leadership centers on an individual who, through motivation, empowers other subordinates to collaborate in building the team. It encompasses idealized influence, inspirational motivation, intellectual stimulation, and individualized consideration [21, 22]. In the healthcare setting, transformational leadership fosters group progression and attainment of higher levels of competence and capability. Multiple studies have found that the antecedents of transformational leadership are strongly linked to job satisfaction for nursing staff, including nurses and nurse assistants [23–25].

Transformational leadership helps reduce burnout in nursing staff by promoting overall health, improving motivation, reducing job stress, and enhancing well-being [26–28]. Transformational leadership creates a collaborative team environment where nursing staff can develop satisfying, supportive relationships and directly engage with their leaders and colleagues [29]. Transformational leadership can foster career growth through mentorship programs, professional development opportunities, and continuous education to deepen their expertise and incorporate strategic financial management into their operational roles [30, 31].

Furthermore, leaders are inclined to provide more opportunities for career growth to their subordinates [32]. The opportunity for career growth has been identified as a critical factor in retaining nurses. This growth opportunity includes work-related learning, development of potential, the chance for advancement, and opportunities for increased influence and authority [17, 33].

Career growth encourages exchanging experiences across different job domains within project teams, and training sessions demonstrate the organization's commitment to continuous change, learning, and career and personal development. Furthermore, effectively managing nurses' development in competitive career advancement planning is essential to leadership [34, 35].

Nursing well-being encompasses mental, physical, and social health while on the job [36]. Nursing is widely recognized as one of the most high-pressure occupations compared to other careers [37, 38]. The scarcity of nurses has increased work hours, placing significant strain on nurses [39]. The heightened work pressure has been found to reduce nurses' job fulfillment, leading to a decline in their commitment to their roles, ultimately resulting in consistently high turnover rates [40–42].

Nurses frequently express worries about their employers' lack of consideration for their requests to improve work-life balance through reduced hours, flexible schedules, and a supportive work environment [43]. The challenge of balancing personal and professional life in the nursing field can result in decreased job satisfaction. More research on work-life balance in the nursing profession needs to be conducted, which has recently gained attention from researchers [44]. Studies have verified a strong connection between work-life balance, intention to leave, and staff retention, underscoring the importance for healthcare organizations to address nurses' work-life issues to enhance their overall well-being [45]. A satisfactory work-life balance contributes to a better work attitude, a strong sense of belonging, and job satisfaction. It increases the likelihood of nurses staying in their roles to support an optimal work environment [46, 47].

The concept of work-life balance involves nurses effectively managing a flexible work schedule to ensure proper time distribution between work and personal life [48, 49]. This study holds significant value as it sheds light on the essential factors influencing nurse retention, particularly the impact of transformational leadership and work-life balance. By pinpointing these critical predictors, the research offers practical guidance for healthcare organizations aiming to enhance nurse retention rates, which is crucial for maintaining high-quality patient care and minimizing costs associated with turnover. Furthermore, decreasing staff turnover and absenteeism markedly enhances nursing staff's well-being and overall health, ultimately leading to improved and more effective healthcare delivery. This, in turn, boosts the efficiency and productivity of healthcare facilities in addressing societal demands.

Moreover, the findings advance the literature on nurse retention, underscoring the necessity for targeted strategies prioritizing leadership practices and work-life balance. This research also paves the way for future investigations into additional factors, such as career growth and work well-being, which could further refine retention strategies. Our research has provided valuable insights for nurses and leaders to address to improve career growth and nursing staff retention within the nursing profession. Therefore, this study examines the impact of transformational leadership, career growth opportunities, work well-being, and work-life balance on nurse retention.

## Research questions

- 1. How does transformational leadership influence nurses' retention in hospitals?
- 2. What is the role of career growth opportunities in affecting nurses' retention rates?
- 3. To what extent does work well-being impact the retention of nurses?
- 4. How does work-life balance influence nurses' decisions to stay in their current roles?
- 5. Which factors, transformational leadership, career growth, work well-being, or work-life balance, significantly impact nurses' retention?

## Methods

### Research design

This study employed a descriptive correlational cross-sectional research design to examine the effects of transformational leadership, career growth opportunities, work well-being, and work-life balance on nurse retention.

### Setting

The research was conducted in the inpatient wards of the main building at Mansoura University Hospital (MMU), a prominent healthcare institution located in Mansoura city, Dakahlia governorate. MMU has a bed capacity of 1,800 and is affiliated with the Ministry of Higher Education. The hospital provides a comprehensive range of healthcare services to meet the needs of the Delta Region, ensuring high-quality care for patients across various inpatient units.

### Subjects, sample size and sampling

The sample size was estimated for this study based on the following formula: n$\frac{(\text{Zi}-\alpha /2)^2.\text{P}(1-\text{P})}{d^2}$, Where Z1 − α/2*Z*1 − *α*/2 (the desired confidence level) corresponds to 5% types 1 error (*p* < 0.05), which is 1.96, based on previous literature [50], the expected proportion (P) in the population was set at 0.468, and the absolute margin of error (d) was defined as 0.061. Plugging these values into the formula yields *n*= $\frac{(1.96)^2.(0.468)(1-0.468)}{(0.061)^2}$  = 257. Thus, the total sample size required for the study was determined to be 257 nurses, which is the total number of potential respondents.

A convenience sampling method was employed to recruit staff nurses responsible for patient care in the inpatient units. Participants were required to meet specific inclusion criteria: they needed to be currently employed as staff nurses at Mansour University Hospital (MMU), be at least 21 years old, be of both genders, and be willing to provide informed consent to participate in the study. Additionally, they must have had at least six months of continuous employment in their current nursing role. Conversely, the exclusion criteria included nurses on leave or not actively engaged in patient care during the study period, those with less than six months of experience in their current position, and individuals who declined to participate or did not provide informed consent.

### Participants recruitment

**Fig. 1:**
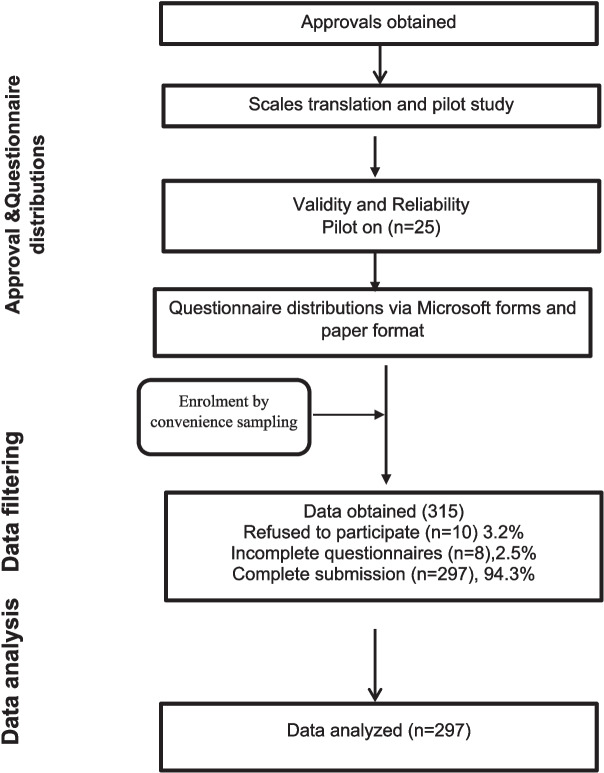
Flow chart for data collection process from nurses

Out of the 315 participants initially invited to participate, 10 nurses (3.2%) declined to participate, while 8 nurses (2.5%) submitted incomplete questionnaires. Consequently, 297 completed questionnaires were collected, representing 94.3% of the initial sample. This resulted in an overall dropout rate of 5.7%. The flow chart detailing this data collection process is illustrated in Fig. 1.

### Study measurements

The study's data was collected using the following five measures and the nurses' Demographic and Clinical Data Questionnaire. All measures were administered in Arabic.

### Nurses demographic and clinical data questionnaire

The questionnaire includes demographic and professional characteristics, explicitly focusing on age, gender, marital status, educational qualifications, years of experience, and current position within the unit.

### Nursing Retention Index (NRI)

The Nursing Retention Index (NRI), developed by Cowin (2002) [51] assesses nurses' intentions to continue in their present jobs or pursue other career opportunities. The NRI comprises six statements—four positive and two negative—assessed on an 8-point Likert scale, with answers ranging from 1 (certainly false) to 8 (definitely true). Each positive statement is scored directly based on the respondent's selection. For negative statements, scores are reverse-coded to maintain consistency. For example, if a respondent rates a negative statement as a "1" (certainly false), it would be counted as "8" (definitely true) in the total score. In statistical analysis, negative items are reverse-scored to ensure consistency. Low Scores (8–24) indicate a weak intention to stay in nursing, and High Scores (37–48) Suggest strong commitment and satisfaction with the nursing profession, indicating a high likelihood of retention. The NRI total score may vary from 8 to 48, with greater values indicating a stronger desire to remain in nursing. The index has strong internal consistency, with Cronbach's alpha of 0.90 reported by Bell and Sheridan, (2020) [52].

### Global Transformational Leadership (GTL) scale

The GTL scale is a seven-item tool developed by Carless et al. in 2000 to evaluate transformational leadership (TL) characteristics in the health and nursing sectors [53, 54]. Participants evaluate each topic using a 5-point Likert scale, with responses ranging from 1 (never) to 5 (nearly usually). The scores for all seven items are summed to produce a total score. Low Scores (7–14) Indicate a weak perception of transformational leadership. High Scores (26–35) Suggest a strong perception of transformational leadership qualities. The total score can range from 7 to 35, with higher scores indicating a stronger perception of transformational leadership qualities. The GTL Scale has remarkable internal consistency, shown by a Cronbach's alpha of 0.94, as reported by Lavoie-Tremblay et al. (2016) [54] and a Cronbach's alpha of 0.91 in the present research. The scale demonstrates robust convergent validity, as indicated by substantial positive correlations with the Leadership Practices Inventory (LPI) scores (*r* = 0.87, *p* < 0.001) and the Multifactor Leadership Questionnaire (MLQ) scores (*r* = 0.79, *p* < 0.001), thereby affirming its efficacy in assessing transformational leadership behaviors [55].

### Career Growth Scale (CGS)

The Career Growth Scale (CGS) is a questionnaire initially established by Weng et al.,(2011) [56], and subsequently revised by Liu et al., (2015) [57, 58]. The assessment has 15 questions, each evaluated on a 5-point Likert scale, with answers ranging from 1 (strongly disagree) to 5 (strongly agree). The CGS has three sub-dimensions: "career goal," "career capacity," and "career opportunity." Each item is scored based on the participant's response, with higher scores reflecting a more positive perception of career growth. The cumulative score for the CGS, which ranges from 15 to 75, is derived by aggregating the values from the three sub-dimensions. Low Scores (15–30) indicate a weak perception of career growth. High Scores (56–75) suggest a strong perception of career growth, indicating confidence in achieving career goals and recognizing ample opportunities for advancement [59]. Examples of items include “My present job is relevant to my career goals and vocational growth” [60]. The first edition of the scale had Cronbach's alpha coefficients for the four sub-dimensions as 0.86, 0.85, 0.80, and 0.78, indicating strong internal consistency [60].

### Well-Being Work Scale (WBWS)

The WBWS, developed by Paschoal and Tamayo (2008) [61], measures three key factors: Positive Affects (PA), Negative Affects (NA), and Emotional Fulfillment (EF), with a total of 30 items. Each item on the scale is evaluated using a 5-point response format. For the first two factors (PA and NA), respondents indicate intensity using the following options: 1 (Not at all), 2 (Not much), 3 (Moderately), 4 (Quite a lot), and 5 (Extremely). For the Emotional Fulfillment factor, an agreement scale is used with options ranging from 1 (Disagree) to 5 (Agree). The scores for each factor are summed separately to derive sub-scores. The cumulative score for the WBWS is calculated by summing the total scores from all three factors, resulting in a total score ranging from 30 to 150. Low Scores (30–60) Indicate low well-being, suggesting high adverse effects and low positive effects and emotional fulfillment. High Scores (106–150) Suggest a strong sense of well-being at work, characterized by high positive effects, low adverse effects, and significant emotional fulfillment [62, 63]. Examples of items include "happy" for Positive Affects, "angry" for Negative Affects, and "I achieve my potential" for Emotional Fulfillment. This structure allows for a comprehensive assessment of well-being in the workplace, capturing both positive and negative emotional experiences and the sense of achieving personal goals. Each factor exhibits high internal consistency, measured by Cronbach's alpha: Positive Affect: α = 0.93*,* Negative Affect: α = 0.91, Achievement/Expressiveness: α = 0.88 [64].

### Work-Life Balance Scale (WLBS)

The WLBS, adapted by Hayman (2005) [65], from Fisher's original study (2001), has 15 questions intended to assess three aspects of work-life balance: Work Interference with Personal Life (WIPL), Personal Life Interference with Work (PLIW), and Work/Personal Life Enhancement (WPLE). The scale has 7 questions for WIPL, which evaluates the influence of work on personal life; 4 items for PLIW, assessing the effect of personal life on work; and 4 items for WPLE, measuring the positive overflow from work to personal life. Participants use a 5-point Likert scale from "Strongly Disagree" (1) to "Strongly Agree" (5), with rising values signifying more development of work/personal life. The comprehensive work-life balance score is derived by aggregating the scores from the three aspects, where elevated values indicate superior work-life balance, primarily via improved personal life experiences resulting from employment. The scores for each dimension are summed separately to derive sub-scores. The cumulative score for the WLBS is calculated by summing the total scores from all three dimensions, resulting in a total score ranging from 15 to 75 [66]. The scale has robust internal consistency, shown by Cronbach's alpha values of 0.91 for WIPL, 0.82 for PLIW, and 0.67 for WPLE [67].

### Validity and reliability of measurements

The NRI, GTL, CGS, and WBWS instruments were carefully translated into Arabic. Bilingual experts fluent in English and Arabic were involved to ensure the translations were accurate and culturally relevant. Two bilingual members of the research team conducted the initial translations. These translations were then back-translated into English by two independent bilingual experts not associated with the research team to verify linguistic consistency and resolve discrepancies. After the translation process, face validity assessments for each instrument were performed with input from seven nursing sciences experts. The feedback from these experts included suggestions for rephrasing certain items to enhance clarity and ensure that the target population would easily understand the terminology. Based on this feedback, minor adjustments were made to improve the clarity and relevance of the questionnaire items.

The Content Validity Index (CVI) was calculated for the Arabic versions of the instruments to evaluate content validity. Experts evaluated each item using a 4-point scale ranging from 1 (not relevant) to 4 (very relevant). The item-level content validity index (I-CVI) was calculated by dividing the number of experts who assessed an item as 3 or 4 by the total number of experts. The scale-level content validity index (S-CVI) was then computed as the mean of the item-level content validity indices (I-CVIs). The resulting CVI scores indicated strong content validity: 0.89 for the NRI, 0.87 for the GTL, 0.91 for the CGS, and 0.86 for the WBWS. Meanwhile, the Arabic version of the WLBS was used**.** Additionally, Cronbach’s alpha values were calculated for each scale: 0.90 for WLBS, 0.89 for GTL, 0.90 for the CGS, 0.89 for the NRI, and 0.89 for the WBWS. These values indicate that all the instruments used are highly reliable.

### Pilot study

Before initiating data collection, pilot research was conducted with 10% of the nursing staff from Mansoura Hospital, including 21 nurses and 4 head nurses. This pilot research sought to evaluate the clarity, comprehensiveness, accessibility, and usefulness of the tools and estimate the necessary time for questionnaire completion. The pilot research sample was excluded from the primary study sample.

### Data collection

The data collection for the study was approved by the Research Ethics Committee of the Faculty of Nursing at Mansoura University, and authorization was secured from both the hospital director and nursing directors. This process took place from September to November 2024. Before data collection, the research team coordinated with the head nurse in each ward to explain the study and secure permission for participant enrollment within their respective care units. Following consent from the nursing directors, researchers distributed hard copies of the questionnaire to potential participants. An online version of the questionnaire was also created on the Microsoft Forms platform, which could be shared via WhatsApp or provided as a hard copy.

The first page of the questionnaire included detailed information about the study’s objectives, data usage, and confidentiality measures. Nurses who agreed to participate must indicate their informed consent by checking a box before accessing the surveys. For the hard copy questionnaire, each respondent completed the form and returned it in person, while the online version was designed to prevent multiple submissions. This ensured that no individual could complete the questionnaire more than once. All completed questionnaires were included in the data analysis, with access restricted solely to the research team. Trained researchers conducted structured interviews during the nurses’ breaks, lasting approximately 10–15 min each. The purpose of these interviews was to verify that all questionnaires were completed correctly.

### Statistical analysis

All statistical analyses were conducted using SPSS for Windows version 24.0 (SPSS, Chicago, IL). The data's normality was evaluated using the Kolmogorov–Smirnov test. Continuous data exhibited a normal distribution and are expressed as mean ± standard deviation (SD), whilst categorical data are represented as counts and percentages. A one-way analysis of variance (ANOVA) was used to compare several groups utilizing continuous data. The Chi-square test was used for categorical data to analyze associations between variables. Furthermore, linear regression analysis was used to ascertain the factors influencing the Nurse Retention Scale. The questionnaires' reliability in the study was assessed by internal consistency testing, with Cronbach's alpha computed for each scale. A criterion for statistical significance was established at *p* < 0.05.

## Results

Table 1 reveals that more than half of the participants (58.9%) were between 25 and 30 years old, with a mean age of 29.8 (3.9) years, 68.4% were females, and most of them were married (68.7%). Participants' positions within their units showed that nearly half (50.2%) were nurses, while nursing technicians accounted for 18.5%. Educational qualifications indicated that a significant majority (63.6%) hold a Bachelor of Nursing degree, with secondary nursing technicians at 21.5% and nursing diploma holders at 12.5%. Experience levels vary among participants, with the largest group (42.8%) having between six and ten years of experience at their current place of employment.

**Table 1:** Distribution of the demographic characteristics of the participants

	**n**	**%**
**Age (Years)**		
25—< 30	175	58.9
30—< 35	96	32.3
35—< 40	14	4.7
40 or More	12	4.0
**M ± SD**	29.8 ± 3.9	
**Gender**		
Male	94	31.6
Female	203	68.4
**Marital Status**		
Single	81	27.3
Married	204	68.7
Divorced / Widowed	12	4.0
**Position in the unit**		
Nurse	149	50.2
Nursing technician	55	18.5
Nursing supervisor	34	11.4
Head of unit	59	19.9
**Educational Qualification**		
Nursing Diploma	37	12.5
Secondary Nursing Technician	64	21.5
Bachelor of Nursing	189	63.6
Master’s or PhD	7	2.4
**Years of Experience at the Current Place**		
1 – 5	121	40.7
6 – 10	127	42.8
11 – 15	33	11.1
16 or More	16	5.4
**M ± SD**	7.3 ± 3.4	

*M* Mean, *SD* Standard Deviation

Table 2 presents the total WLB score of 43.7 with a standard deviation (SD) of 6.3. The three subscales yielded the following average scores: the work interference with personal life had a mean of 22.6 (SD = 5.1), the work/personal life enhancement aspect scored an average of 11.4 (SD = 3.0), and personal life interference with work had a mean score of 9.7 (SD = 2.9). The GTL Scale revealed a total score of 18.9 (SD = 6.9). The idealized influence and inspirational motivation dimensions scored 5.4 (SD = 2.1 and SD = 2.3, respectively). Individual consideration scored 5.3 (SD = 2.1), while intellectual stimulation scored 2.7 (SD = 1.2). For the CGS, the total score was 48.2 (SD = 11.1). Within this, career goals averaged 13.3 (SD = 3.4), career capacity was at 14.6 (SD = 3.4), and career opportunities scored an average of 20.3 (SD = 5.7). The WBWS indicated a total score of 48.2 (SD = 11.1). The average scores for expressiveness and fulfillment at work were 21.9 (SD = 7.4), and positive affect was 23.5 (SD = 6.4). However, the negative affect had a mean score of 34.2 (SD = 12.5). Finally, NRIS indicated an average score of 30.8 (SD = 6.6).

**Table 2:** The mean scores of the studied variables

Variables	M (SD)
**WLB Scale**	
Work Interference with Personal Life	22.6 (5.1)
Work/Personal Life Enhancement	11.4 (3.0)
Personal Life Interference with Work	9.7 (2.9)
Total of WLB	43.7 (6.3)
**GTL Scale**	
Idealized Influence	5.4 (2.1)
Inspirational Motivation	5.4 (2.3)
Individual Consideration	5.3 (2.1)
Intellectual Stimulation	2.7 (1.2)
Total of GTL Score	18.9 (6.9)
**CGS**	
Career Goal	13.3 (3.4)
Career Capacity	14.6 (3.4)
Career Opportunity	20.3 (5.7)
Total of CGS	48.2 (11.1)
**WBWS**	
Expressiveness/Fulfillment at Work (EF)	21.9 (7.4)
Positive Affect (PA)	23.5 (6.4)
Negative Affect (NA)	34.2 (12.5)
Total of WBWS	48.2 (11.1)
NRIS	30.8 (6.6)

*M* Mean, *SD* Standard Deviation, *WLB* Work-Life Balance Scale, *GTL* Global Transformational Leadership, *CGS* Career Growth Scale, *WBWS* Work Well-Being Scale, *NRIS* Nursing Retention Index Scale

**Fig. 2:**
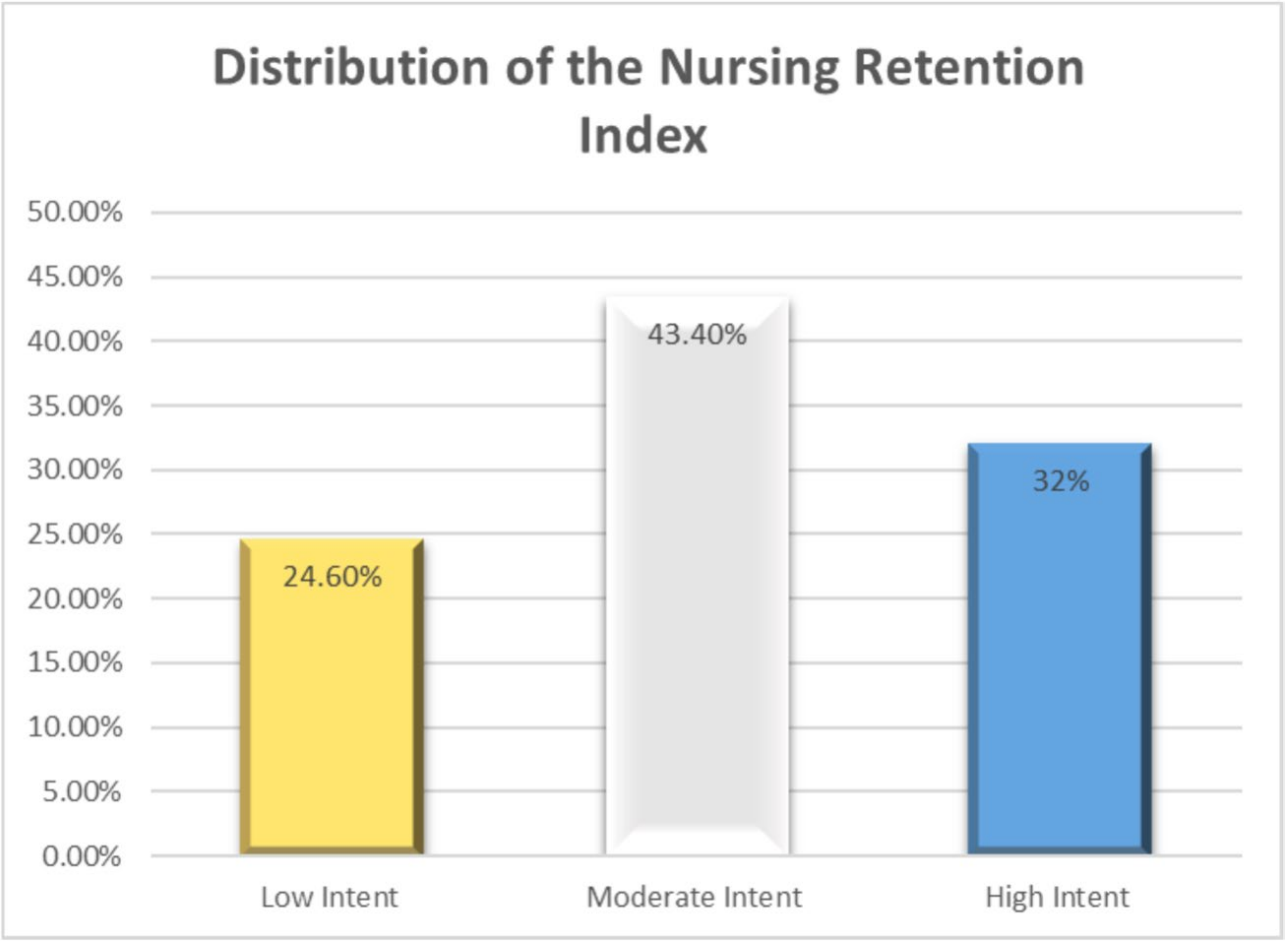
Distribution of the nursing retention index

Figure 2 illustrates the distribution of the NRI within healthcare settings. It showed that 43.4% of the participants had a moderate intent to retain, 32.0% had a high level, and only 24.60 had a low intention to retain within their settings.

Table 3 shows that age significantly impacted the WLBS and CGS scores. Participants aged 25 to 30 reported the highest mean WLBS score of 44.5 (SD = 6.4), with a statistically significant difference (F = 2.830, *P* = 0.039). For CGS, participants aged 35 to 40 showed the highest mean score of 54.4 (SD = 10.5), indicating significant differences (F = 3.630, *P* = 0.013). Additionally, there was a strong association between age and WBWS scores, with younger participants reporting higher scores (F = 6.578, *P* < 0.001). Marital status significantly influenced WLBS and NRIS scores. Single participants had the highest WLBS mean score of 44.8 (SD = 6.1), with a notable difference (F = 6.521, *P* = 0.002). For NRIS, divorced or widowed participants had lower retention scores (29.3 ± 4.4) compared to married individuals (31.5 ± 6.3), indicating a significant relationship (F = 3.652, *P* = 0.027). The educational qualification also showed substantial associations with WLBS and GTLS scores; those with a Master’s or PhD reported the highest GTLS mean score of 26.3 (SD = 7.7) and a CGS mean score of 53.6 (SD = 12.2) compared to other educational levels (F = 3.029, *P* = 0.030 for GTLS and F = 4.060, *P* = 0.008 for WLBS). Lastly, years of experience at the current place of employment affected WLBS and NRIS scores significantly; those with one to five years reported the highest WLBS mean score of 44.8 (SD = 6.1), (F = 4.261, *P* = 0.006). For NRIS, participants with sixteen years or more had higher retention scores than those with fewer years of experience (34.6 ± 5.0), with significance noted at F = 3.778, *P* = 0.011.

**Table 3:** Association between the demographic characteristics of the participants with work-life balance, global transformational leadership, career growth scale, nursing retention index, and work well-being scale

	WLBS	GTLS	CGS	NRIS	WBWS
	Mean ± SD	Mean ± SD	Mean ± SD	Mean ± SD	Mean ± SD
**Age (Years)**					
25—< 30	44.5 ± 6.4	18.9 ± 7.1	47.4 ± 11.3	30.4 ± 6.9	82.0 ± 13.9
30—< 35	42.6 ± 6.2	18.2 ± 6.2	47.8 ± 10.4	31.1 ± 6.5	74.7 ± 12.9
35—< 40	43.4 ± 5.2	21.8 ± 9.5	54.4 ± 10.5	31.8 ± 4.8	83.7 ± 8.7
40 or More	40.6 ± 4.6	21.3 ± 6.4	55.4 ± 10.1	34.2 ± 5.3	79.5 ± 12.7
	F = 2.830, *P* = 0.039*	F = 1.567, *P* = 0.197	F = 3.630, *P* = 0.013*	F = 1.477, *P* = 0.221	F = 6.578, *P* < 0.001**
**Gender**					
Male	43.6 ± 6.4	19.2 ± 6.8	46.9 ± 11.8	30.4 ± 6.9	81.1 ± 13.2
Female	43.7 ± 6.2	18.8 ± 7.1	48.8 ± 10.7	31.0 ± 6.5	78.9 ± 14.0
	t = 0.048, *P* = 0.962	T = 0.438, *P* = 0.662	T = 1.370, *P* = 0.172	T = 0.801, *P* = 0.424	T = 1.274, *P* = 0.204
**Marital status**					
Single	44.8 ± 6.1	18.8 ± 7.1	46.6 ± 10.9	29.3 ± 7.5	81.6 ± 13.2
Married	43.5 ± 6.2	18.9 ± 6.9	48.6 ± 10.8	31.5 ± 6.3	78.7 ± 14.1
Divorced / Widowed	38.0 ± 5.4	19.2 ± 8.3	51.4 ± 15.7	29.3 ± 4.4	80.8 ± 9.5
	F = 6.521, *P* = 0.002*	F = 0.018, *P* = 0.983	F = 1.538, *P* = 0.216	F = 3.652, *P* = 0.027*	F = 1.300, *P* = 0.274
**Position in the unit**					
Nurse	44.2 ± 5.9	18.0 ± 6.7	48.1 ± 9.9	30.6 ± 6.3	79.6 ± 12.7
Nursing technician	43.3 ± 5.9	19.2 ± 6.5	49.2 ± 11.5	30.9 ± 6.1	78.7 ± 17.6
Nursing supervisor	40.7 ± 7.3	20.4 ± 7.2	48.5 ± 10.6	30.7 ± 7.5	77.8 ± 14.0
Head of unit	44.4 ± 6.5	20.1 ± 7.7	47.2 ± 13.7	31.3 ± 7.4	81.5 ± 12.0
	F = 3.186, *P* = 0.024*	F = 1.997, *P* = 0.114	F = 0.337, *P* = 0.799	F = 0.157, *P* = 0.925	F = 0.658, *P* = 0.579
**Educational Qualification**					
Nursing Diploma	40.9 ± 7.4	18.0 ± 7.6	47.0 ± 12.8	30.5 ± 7.6	75.8 ± 12.5
Secondary Nursing Technician	42.8 ± 4.7	18.3 ± 6.9	47.4 ± 11.0	31.8 ± 5.4	82.1 ± 12.8
Bachelor of Nursing	44.5 ± 6.3	19.0 ± 6.7	48.5 ± 10.7	30.5 ± 6.8	79.5 ± 14.3
Master’s or Doctorate	43.6 ± 8.1	26.3 ± 7.7	53.6 ± 12.2	33.0 ± 6.2	80.4 ± 7.1
	F = 4.060, *P* = 0.008*	F = 3.029, *P* = 0.030*	F = 0.835, *P* = 0.476	F = 0.878, *P* = 0.453	F = 1.683, *P* = 0.171
**Years of Experience at the Current Place**					
1 – 5	44.8 ± 6.1	19.0 ± 7.3	47.2 ± 11.9	29.5 ± 7.2	81.6 ± 15.0
6 – 10	43.5 ± 6.3	18.7 ± 6.4	47.9 ± 10.0	31.5 ± 6.1	78.8 ± 13.3
11 – 15	41.8 ± 6.5	18.0 ± 7.7	49.1 ± 11.2	31.3 ± 6.4	76.3 ± 10.2
16 or More	40.1 ± 4.8	21.6 ± 7.4	55.8 ± 10.5	34.6 ± 5.0	78.1 ± 12.6
	F = 4.261, *P* = 0.006*	F = 1.062, *P* = 0.366	F = 2.948, *P* = 0.033*	F = 3.778, *P* = 0.011*	F = 1.694, *P* = 0.168

*M* Mean, *SD* Standard Deviation, *WLBS* Work-Life Balance Scale, *GTLS* Global Transformational Leadership Scale, *CGS* Career Growth Scale, *WBWS* Work Well-Being Scale, *NRIS* Nursing Retention Index Scale

F: One-way ANOVA; t: Student’s t-test

^*^Significant at the < 0.05 level

^**^Significant at the < 0.001

Table 4 presents that the WLBS had a significant positive correlation with the GTLS, CGS, and NRIS with coefficients of *r* = 0.154, 0.130, and 0.288, respectively (*p* = 0.003, 0.025, 0.001). Additionally, GTLS significantly correlated with CGS, NRIS, and WBWS (*r* = 0.590, 0.247, and 0.209, *p* < 0.001). CGS demonstrated a strong positive correlation with NRIS (*r* = 0.456, *p* < 0.001) and with the WBWS (*r* = 0.268, *p* < 0.001). Lastly, NRIS and WBWS were significantly positively correlated (*r* = 0.257, *p* < 0.001).

**Table 4:** Correlation coefficient between work-life balance, global transformational leadership, career growth, nursing retention, and work well-being

	**WLBS**		**GTLS**		**CGS**		**NRIS**		**WBWS**	
	**r**	**P**	**r**	**p**	**r**	**p**	**r**	**p**	**r**	**p**
**WLBS**			0.154	0.003*	0.130	0.025*	0.288	< 0.001**	0.248	< 0.001**
**GTLS**	0.154	0.003*			0.590	< 0.001**	0.247	< 0.001**	0.209	< 0.001**
**CGS**	0.130	0.025*	0.590	< 0.001**			0.456	< 0.001**	0.268	< 0.001**
**NRIS**	0.288	< 0.001**	0.247	< 0.001**	0.456	< 0.001**			0.257	< 0.001**
**WBWS**	0.248	< 0.001**	0.209	< 0.001**	0.268	< 0.001**	0.257	< 0.001**		

^*^Correlation is significant at the 0.05 level (2-tailed)

^**^Correlation is significant at the 0.01 level (2-tailed)

*WLBS* Work-Life Balance Scale, *GTLS* Global Transformational Leadership Scale, *CGS* Career Growth Scale, *WBWS* Work Well-Being Scale, *NRIS* Nursing Retention Index Scale

Table 5 reveals that the WLBS was a significant predictor of nurse retention, with an unstandardized coefficient of 0.255 (*p* < 0.001), indicating that the retention index increases by approximately 0.255 units for every unit increase in work-life balance. A standardized coefficient (Beta) of 0.426 further supported this positive relationship. GTLS also emerges as a significant predictor, with an unstandardized coefficient of 0.082 (*p* = 0.002) and a standardized coefficient of 0.171. In contrast, the CGS and WBWS did not significantly predict retention; CGS had an unstandardized coefficient of -0.082 (*p* = 0.154), while WBWS showed an unstandardized coefficient of -0.042 (*p* = 0.482). The overall model demonstrated an R-squared value of 0.234, meaning that approximately 23.4% of the variance in nurse retention can be explained by the independent variables included in the analysis with significant F-statistic (F = 22.294, *p* < 0.001), indicating that the model is statistically significant.

**Table 5:** Linear regression analysis on factors predicting nurses retention

	**Unstandardized Coefficients**		**Standardized Coefficients**	**T**	**Sig**	**F**	**Sig**	**95% CI**	
	**B**	**Std. Error**	**Beta**					***LL***	***UL***
(Constant)	16.369	3.121		5.245	0.000**	22.294	0.000**		
WLBS	0.255	0.039	0.426	6.497	0.000**			0.211	0.514
GTLS	0.082	0.027	0.171	3.066	0.002**			0.020	0.231
CGS	-0.082	0.057	-0.077	-1.429	0.154			-0.035	-0.123
WBWS	-0.042	0.060	-0.045	-0.703	0.482			-0.012	-0.143
*R* = *0.484, R – Square* = *0.234, Adjusted R – Square* = *0.223*									

*WLBS* Work-Life Balance Scale, *GTLS* Global Transformational Leadership Scale, *CGS* Career Growth Scale, *WBWS* Work Well-Being Scale (independent variables, *NRIS* Nursing Retention Index Scale (Dependent variable)

F: ANOVA test

*R*^2^: Coefficient of determination

*CI* confidence Interval

B: Unstandardized Coefficients

Beta: Standardized Coefficients

*t* t-test of significance

*LL* Lower limit, *UL* Upper Limit

^**^Statistically significant at *p* ≤ 0.01

## Discussion

Egypt's healthcare sector is experiencing a significant shortage of qualified nurses, threatening the quality and accessibility of healthcare services. High turnover rates, driven by low wages, heavy workloads, and limited professional development, exacerbate this issue. Addressing nurse retention is essential with rising healthcare demands due to Egypt's growing population. This study investigated the impact of transformational leadership, career growth, work well-being, and work-life balance on nurse retention.

Our findings revealed that the NRIS recorded an average score of 30.8 (SD = 6.6), indicating moderate retention among most nurses. Participants experienced a balanced yet moderately variable relationship between work and personal life, with a total Work-Life Balance (WLB) score of 43.7 (SD = 6.3). Work interference with personal life had a mean score of 22.6 (SD = 5.1), while personal life interference with work averaged 9.7 (SD = 2.9). Leadership perceptions were moderate, with a total GTL score of 18.9 (SD = 6.9), and career aspirations were well-balanced, with a total CGS score of 48.2 (SD = 11.1). Emotional well-being showed variation, reflected by a WBWS total score of 48.2 (SD = 11.1), highlighting a mix of positive and negative experiences.

This could be that salary benefits, staffing numbers, administrative workloads, and the accessibility of necessary supplies are some variables that influence nurse retention. The quality of relationships with patients, their families, and co-workers significantly impacts retention, as does the extent to which nurses feel independent and empowered in their decision-making regarding patient care. Nurses are more likely to stay in their positions when they have decision-making authority because it increases their professional fulfilment and job satisfaction. Similarly, a 2018 cohort study involving 21,050 newly hired nurses from 304 hospitals in South Korea tracked turnover rates and risk factors over 18 months. The study revealed that 26.4% of nurses left within their first year, with 20.1% resigning in the first 6 months and 6.3% between 7 and 12 months. The turnover risk was higher with an increased bed-to-nurse ratio, suggesting that staffing levels and workload significantly influence nurse retention [68]. These findings contrast with previous research conducted at Menoufia University Hospital in Shebin-ELkom, which involved 400 nurses. The results demonstrated that more than half of the nurses exhibited low levels of work engagement and held a negative perception of talent management. Furthermore, the majority of nurses experienced poor retention rates. Overall, the study established a strong positive relationship between nurses' retention, work engagement, and perceptions of talent management [69].

Concerning socio-demographic factors, our outcomes indicate that age, marital status, education, and years of experience influenced WLBS scores and CGS. Younger nurses reported higher WLBS and CGS scores. Single nurses had better WLBS scores, while divorced or widowed nurses showed lower retention. Higher education levels, particularly Master's or Doctorate degrees, were linked to better WLBS and career growth. Nurses with 16 or more years of experience at their current workplace also had higher retention. These findings may be due to higher education enhancing knowledge, skills, and career advancement opportunities. Nurses with advanced degrees, such as a Master's or Doctorate, often experience greater job satisfaction, confidence, and work-life balance. Their qualifications offer more career flexibility, leading to higher advancement opportunities and a more manageable workload. These findings align with Garside's (2023) [70] systematic review, which emphasized that work-life balance is crucial for nurses at all career stages. Nurses consistently seek flexibility, family-friendly policies, fair compensation, and opportunities for professional development. Notably, the most satisfied nurses tend to have more experience and have recently taken on new roles. This highlights the importance of retaining nurses in the profession and suggests that keeping them in the same position when dissatisfied can be counterproductive if they feel disengaged. Similarly, de Vries et al. (2023) [71] found that nurses with more experience are likelier to remain in their roles, with retention being incredibly high among older nurses, particularly those in middle age or beyond.

The study found that better WLBS is positively linked to favourable leadership perceptions GTLS, career growth CGS, retention NRIS, and well-being WBWS. Effective leadership GTLS was positively linked to career growth and retention, while CGS positively correlates with higher retention and job satisfaction. Additionally, higher retention of NRIS is associated with improved well-being of WBWS. This can be attributed to leadership styles, coping abilities, and personal traits influencing nurses' retention. Transformational leadership is vital in improving retention by empowering, supporting, and inspiring nurses. It creates an encouraging work environment that enhances long-term commitment, reduces burnout, and boosts job satisfaction. Variations in nurses' retention rates across hospitals can often be attributed to the leadership style employed by hospital administrators. Transformational leaders address individual needs, build trust, and help reduce turnover, resulting in a more stable and committed nursing workforce.

Numerous studies supported these findings, including Farahani et al. (2024) [72], which identified six key factors influencing nurse retention: personal traits, job needs, employment services, working conditions, workplace relationships, and organizational culture. Work-life balance, career advancement, and job satisfaction were the primary factors affecting retention. While there are similarities, there are differences between retention factors in European and non-European countries, highlighting the need for multifactorial approaches to develop effective retention strategies. Thirty-four studies found that nurses stay because they are satisfied with their jobs and devoted to their organizations. The factors that permeate these notions weigh differently across generations. While not a perfect explanation, they show substantial disparities in workplace needs by age, influencing intention to stay, job satisfaction, organizational commitment, and, ultimately, nurse turnover [70]. Rony et al. (2023) [73] studied the factors affecting work-life balance in 656 nurses in Dhaka, Bangladesh, finding a positive link between work-life imbalance, unhappiness, and adverse effects on family life. They concluded that supporting work-life balance is crucial for improving healthcare productivity, patient care, and clinical outcomes. Marufu et al. (2021) [17] identified nine domains impacting staff turnover in 47 studies, including leadership, career advancement, organizational environment, staffing, and compensation. De Vries et al. (2023) [71] identified six determinants influencing job retention: organizational culture, working environment, relationships, job demands, employment services, and personal traits. Career advancement, WLB, and job satisfaction significantly influence retention. While European and non-European countries share some similarities, they also show differences in retention factors. According to Sani & Adisa (2024), leadership, mainly through transactional and transformational styles, is crucial for WLB, fostering trust-based, reciprocal exchanges [74].

Similarly, Hashish & Ashour (2020) [75] found that non-monetary strategies by nursing leaders in Egypt are essential for reducing brain drain, with economic and workplace conditions being the most impactful. Yarbrough et al. (2016) [76] highlighted that career growth and professional values are strongly linked to retention and positively correlated with career development and job satisfaction. The integrative review emphasizes that effective leadership cultivates a positive safety culture, eliminating barriers to care, and is crucial for patient safety. Leadership, a positive work environment, job satisfaction, and fewer unpleasant work experiences are all crucial factors in nurse retention. Nurse supervisors foster motivation by creating supportive environments, and specific leadership approaches have been shown to reduce psychological stress, absenteeism, and turnover, ultimately enhancing job satisfaction [77]. The multidimensional theory of commitment highlights that employee commitment, including affective, continuance, and normative components, strongly correlates with retention. Notably, normative commitment (staying out of obligation) has a more significant impact on retention than affective commitment (emotional attachment). Therefore, management should foster affective and normative commitment, providing essential tools and a supportive work environment to enhance employee engagement and retention [78].

Our regression analysis demonstrated that work-life balance and transformational leadership are significant predictors of nurse retention. Specifically, work-life balance had a strong positive influence, with a 0.255 increase in retention for each unit improvement and a high standardized coefficient (Beta = 0.426), highlighting its substantial role in predicting nurses' decisions to remain in their positions. Transformational leadership also positively impacted retention, although to a lesser extent, with a 0.082 increase in retention for each unit increase (*p* = 0.002). Conversely, caregiver Group Support and Workplace Bullying Witnessed did not significantly affect retention statistically. Overall, the model accounted for 23.4% of the variance in nurse retention, suggesting that enhancing work-life balance and leadership practices represents a promising strategy for improving nurse retention rates. The supportive work-life balance might explain how these findings reduce stress and burnout, increasing job satisfaction and retention. Transformational leadership also plays a crucial role in fostering support, inspiration, and professional development. Leaders who communicate well, encourage collaboration, and recognize staff contributions boost job satisfaction, empowerment, and commitment, ultimately enhancing nurse retention and strengthening the healthcare system. A cross-sectional study at King Faisal University's Hospital in Al-Khobar, Saudi Arabia, with 499 nurses, revealed that leadership styles and the availability of challenging opportunities significantly influenced retention, as indicated by ordinal regression analysis. These findings highlight the critical role of leadership and career development in enhancing nurse retention [79].

In this regard, Abdul Salam et al., [80] found a moderate correlation between resilience scores and perceptions of transformational leadership, with leadership perceptions explaining 29% of the variance in resilience. Transformational leadership emerged as a significant predictor of resilience among nurses. Additionally, Sitompul et al., (2024) [81] confirmed a positive linear relationship between work-life balance, work participation, and innovative work behavior [81]. The regression analysis showed that this relationship was significant, with an *R*^2^ value of 0.291. This emphasizes the importance of supporting work-life balance and employee engagement to foster innovation.

## Limitations

While the study presents valuable insights, it is not without its limitations. Firstly, the reliance on hard copies may hinder the generalizability of the findings, as it restricts participation to specific care units and introduces logistical challenges. However, this method does ensure direct verification of respondents' identities, reducing the risk of duplicate responses. Additionally, online questionnaires may limit participation from individuals who lack internet access or are less familiar with digital tools, potentially skewing the sample.

Secondly, the cross-sectional design constrains the ability to establish causal relationships between the variables; a longitudinal approach could provide deeper insights into how these factors influence retention over time. Furthermore, the data relies on self-reported measures, which may introduce bias from participants' subjective perceptions and the possibility of social desirability bias. The study employed simple sampling methods, which could lead to researcher bias and may overlook certain variables related to work circumstances and leadership styles across diverse healthcare settings, ultimately limiting the generalizability of the results to other nursing contexts. Consequently, further research is warranted to address these limitations and enhance our understanding of the dynamics of nurse retention.

## Conclusions

The results reveal moderate work-life balance challenges for participants, with work demands often interfering with personal life. Transformational leadership is positively perceived, though intellectual stimulation is lower. Participants feel they have promising career opportunities but moderate motivation for advancement. Emotional experiences indicate high levels of workplace stress. Younger, single, and highly educated individuals report better work-life balance and retention intentions. Correlations suggest that work-life solid balance and transformational leadership positively influence retention and well-being. Regression analysis confirms that work-life balance and transformational leadership are significant predictors of nurse retention.

## Nursing implications

Transformational leadership is viewed positively, yet there is a noted deficiency in intellectual stimulation, suggesting that leadership styles could be enhanced to better support staff. While nurses recognize promising career opportunities, their motivation for advancement remains moderate, indicating a need for targeted interventions to boost engagement and career development. The high levels of workplace stress reported by participants underscore the necessity for effective stress management strategies within the nursing environment. Interestingly, younger, single, highly educated nurses report better work-life balance and retention intentions, suggesting that demographic factors affect how work-life balance is perceived and managed. The positive correlations between work-life balance, transformational leadership, and nurse retention emphasize the importance of fostering a supportive workplace culture prioritizing employee well-being. To improve retention rates and overall job satisfaction among nurses, healthcare organizations should consider implementing policies that promote work-life balance, such as flexible scheduling and opportunities for professional development. Training programs focused on transformational leadership could also enhance managerial support and create a more stimulating work environment.

## Supplementary Information

Supplementary Material 1.

## Data Availability

The datasets used during the study are available from the corresponding author upon request.
